# Epitaxial Stabilization of Single-Crystal Multiferroic YCrO_3_ Thin Films

**DOI:** 10.3390/nano10102085

**Published:** 2020-10-21

**Authors:** Yogesh Sharma, Elizabeth Skoropata, Binod Paudel, Kyeong Tae Kang, Dmitry Yarotski, T. Zac Ward, Aiping Chen

**Affiliations:** 1 Center for Integrated Nanotechnologies (CINT), Los Alamos National Laboratory, Los Alamos, NM 87545, USA; binodpaudel52@gmail.com (B.P.); ktkang@lanl.gov (K.T.K.); dzmitry@lanl.gov (D.Y.); apchen@lanl.gov (A.C.); 2 Materials Science and Technology Division, Oak Ridge National Laboratory, Oak Ridge, TN 37831, USA; eskoropata@gmail.com (E.S.); wardtz@ornl.gov (T.Z.W.)

**Keywords:** thin films, rare-earth chromites, dielectrics, magnetism, multiferroics

## Abstract

We report on the growth of stoichiometric, single-crystal YCrO_3_ epitaxial thin films on (001) SrTiO_3_ substrates using pulsed laser deposition. X-ray diffraction and atomic force microscopy reveal that the films grew in a layer-by-layer fashion with excellent crystallinity and atomically smooth surfaces. Magnetization measurements demonstrate that the material is ferromagnetic below 144 K. The temperature dependence of dielectric permittivity shows a characteristic relaxor-ferroelectric behavior at *T*_C_ = 375–408 K. A dielectric anomaly at the magnetic transition temperature indicates a close correlation between magnetic and electric order parameters in these multiferroic YCrO_3_ films. These findings provide guidance to synthesize rare-earth, chromite-based multifunctional heterostructures and build a foundation for future studies on the understanding of magnetoelectric effects in similar material systems.

## 1. Introduction

Magnetoelectric multiferroics have emerged as an attractive material system due to their multifunctionality for a variety of potential device applications in energy and memory technologies [1,2,3]. These devices are mainly based on epitaxial thin-film heterostructures with smooth surfaces and interfaces at atomic scale [2,4,5,6,7]. Recent advances in epitaxial growth techniques have enabled the synthesis and design of multiferroic heterostructures that give rise to a variety of multifunctional properties [8,9,10,11,12]. Despite the rapidly growing interest in these materials, access to new multiferroics and their high-quality heterostructures is still limited [2,13,14].

The perovskite rare-earth chromites, *R*CrO_3_ (*R*=rare-earth), are of current interest as a promising system exhibiting multiferroicity and a broad range of other properties, such as spin reorientation transition, multiband optical transitions, exchange bias, and strong spin–phonon interactions [15,16,17,18,19,20,21,22,23,24,25,26]. Among this family of perovskite, YCrO_3_ (YCO, hereafter) has been reported as an insulating antiferromagnet, which shows intriguing functional properties [17,27,28,29]. Recently, YCO has regained interest by showing a canted antiferromagnetic transition at 140 K, resulting in weak ferromagnetism, and a ferroelectric transition at around 473 K, which renders it a multiferroic material [30,31]. Understanding the physical properties of epitaxial YCO is thus of vital importance to explore multifunctionalities for next-generation applications, which demand structurally and compositionally well-defined, high-quality single-crystal thin films. Pulsed laser deposition (PLD) has been utilized to synthesize single-phase YCO epitaxial films [32]. However, it was found to be rather challenging to obtain the desired stoichiometry of YCO in the as-deposited thin films, due to the formation of the oxygen-rich secondary phase (YCrO_3+x_) [32]. An additional post-annealing step was required to completely remove the secondary phases in order to stabilize phase-pure, stoichiometric YCO epitaxial thin films [32]. Moreover, the discussion was mainly limited to the electronic properties and authors did not reveal the multiferroic behavior of the epitaxial YCO films [32].

In this work, we report on the epitaxial growth and physical properties of stoichiometric, multiferroic YCO films, which shows both relaxor-ferroelectric and ferromagnetic orders coexisting in the thin film. We show that the highly crystalline, phase-pure epitaxial YCO thin films can be grown on (001) SrTiO_3_ (STO) substrates using PLD, avoiding any post-synthesis annealing steps. The presence of ferromagnetic ordering below 144 K and a relaxor-ferroelectric-like phase transition at ≈375–408 K, indicate the multiferroic nature of our epitaxial YCO films. In addition, the observation of a dielectric anomaly at the magnetic ordering temperature and the multiband optical absorption properties further exhibit the excellent functionalities of epitaxial YCO films.

## 2. Materials and Methods

A ceramic target of stoichiometric YCO was synthesized using the conventional solid-state reaction method. YCO thin films were then grown using PLD on 5 × 5 × 0.5 mm^3^ atomically flat, TiO_2_-terminated STO (001). A KrF excimer laser (λ = 248 nm) operating at a repetition rate of 5 Hz with a laser fluence of 1.25 J/cm^2^ was used for target ablation. The target–substrate distance was set at 5 cm. Deposition optimization was performed, and an oxygen partial pressure of 20 mTorr at a substrate temperature of 800 °C were the optimal growth conditions. It was found that the growth temperature and oxygen partial pressure play an important role in stabilizing single-phase epitaxial YCO films. After deposition, the films were cooled to room temperature maintaining a 20-mTorr oxygen pressure. The growth rate was approximately 0.08 Å/pulse. For dielectric measurements, platinum (Pt) circular top electrodes (thickness of ~50 nm and diameter of ~200 µm) were deposited on YCO/Nb:STO (001) films using magnetron sputtering. The crystal structure and growth orientation of the films were characterized by X-ray diffraction (XRD) using a Panalytical X’Pert Pro four-circle high resolution X-ray diffractometer with Cu Kα1 radiation. Atomic force microscopy (Nanoscope III AFM) was used in tapping mode to characterize the surface morphology of as-grown films. The X-ray absorption spectroscopy (XAS) measurements were performed at beamline 4ID-C of the Advanced Photon Source at Argonne National Laboratory. SQUID magnetometry was conducted with a Quantum Design MPMS3. The temperature-dependence of the dielectric properties were measured using an LCR meter (HP4284A) with a fixed applied *ac* voltage of 50 mV. The spectroscopic ellipsometry (M-2000, J. A. Woollam Co., Lincoln, NE, USA) was used to obtain the optical conductivity of the film as a function of photon energy at room temperature.

## 3. Results and Discussions

**Figure 1:**
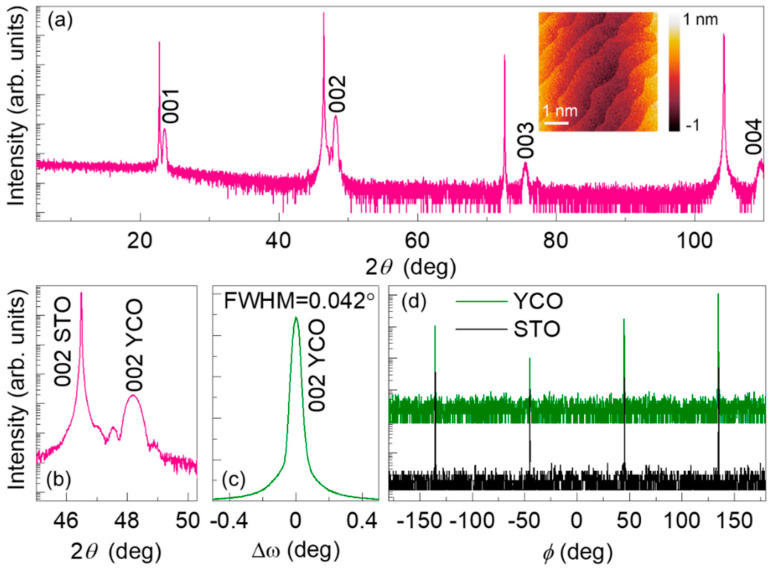
(**a**) Room temperature XRD *θ*–2*θ* line scan of the 32-nm YCrO_3_ (YCO) film grown on SrTiO_3_ (STO) (001) substrate. Inset shows the atomic force microscopy (AFM) image of the same sample. (**b**) The *θ*–2*θ* local scan around the substrate (002) peak. (**c**) A rocking curve of the YCO (002) peak with a full width at half maximum (FWHM) of 0.042° for the 32-nm YCO film. (**d**) *ϕ*-scans of the {222} reflections of film and substrate.

Figure 1a,b show *θ*-2*θ* XRD scans for a YCO film deposited at optimized growth conditions on an STO substrate. XRD scans show that the as-grown film is *c*-axis-oriented epitaxial film with no evidence of secondary phases. The well-defined Laue oscillations around the YCO (002) Bragg peak (Figure 1b) and the periodic oscillations arising from the interfacial interference in the X-ray reflectivity measurement (not shown), indicate high crystallinity with homogeneous, atomically flat surfaces and interfaces of the as-grown 32-nm epitaxial thin film. The AFM image shows the surface morphologies of the film to present a step-and-terrace structure with an atomically smooth surface, indicating the layer-by-layer growth of YCO on STO (inset of Figure 1a). Further evidence of the crystallographic quality was seen as a narrow peak in the 002 rocking curve with a full width at half maximum (FWHM) of 0.042°, as shown in Figure 1c. The FWHM of the substrate (STO) 002 peak was 0.010° (not shown). In the bulk, perovskite YCO exhibits an orthorhombic structure (space group *Pbnm*) with lattice parameters *a* = 5.255 Å, *b* = 5.520 Å, and *c* = 7.536 Å [33]. The pseudocubic unit-cell within the orthorhombic structure yields $a_{pc}\;$= 3.786 Å [31], and the lattice mismatch between YCO and cubic STO (c = 3.905) is around −3.05%. The in-plane epitaxial orientation relationship of pseudocubic YCO on STO is confirmed with the *ϕ*-scans: (001) YCO || (001) STO and [100] YCO || [100] STO (Figure 1d).

**Figure 2:**
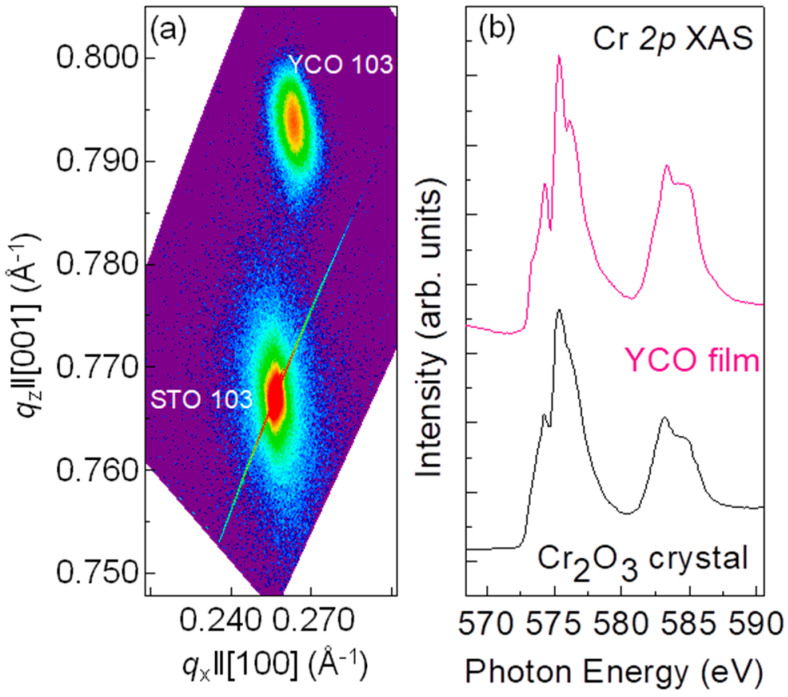
(**a**) Reciprocal space map (RSM) of asymmetric scans around the (103) reflection of STO for the 32-nm YCO film. (**b**) Comparison of the Cr 2*p* XAS spectrum of the YCO film to that of Cr_2_O_3_ in Ref. [36].

To inspect the strain state of the YCO film, reciprocal space mapping (RSM) measurements were performed around the asymmetric (103) Bragg’s reflection of the film and substrate (Figure 2a). The lack of vertical alignment of (103) film with respect to the substrate peak indicates that the film is partially relaxed from the substrate. By calculating lattice parameters from RSM, the in-plane and out-of-plane lattice constants are determined as $a_{∥}$ = 3.801 Å and $a_{⊥\;}$= 3.778 Å for the 32-nm YCO film. The in-plane strain *ε_xx_* = 0.396% and the out-of-plane strain *ε_zz_* = −0.211%, were calculated using *ε_xx_* = ($a_{∥}-a_{pc}$)$/a_{pc}$ and *ε_zz_* = ($a_{⊥}-a_{pc}$)$/a_{pc}$ [34]. The Poisson ratio [*ν* = *ε_zz_*_/_(*ε_zz_* − 2*ε_xx_*)] of the YCO film was calculated to be 0.21, which is close to the value of 0.23, reported for LaCrO_3_ (LCO) films grown by molecular beam epitaxy (MBE) [35].

It has been reported that the Cr^3+^ ions can be oxidized to a higher charge state in the near-surface region of chromites thin films [32,35]. In order to examine the stoichiometry of YCO films, Cr L_2,3_-edge XAS measurements were performed in surface-sensitive total electron yield (TEY) detection mode. Figure 2 shows XAS around the Cr L-edges for the YCO film measured at room temperature. A room temperature XAS spectrum of bulk Cr_2_O_3_ single crystal is shown for reference [36]. A direct comparison between the spectral shapes of Cr in both film and standard bulk Cr_2_O_3_ spectra confirms that there is no obvious deviation from Cr^3+^ which means oxygen stoichiometry is nominal. This is a significant point as previous reports showed oxygen-rich secondary phases of YCrO_3+__ẟ_ [32].

**Figure 3:**
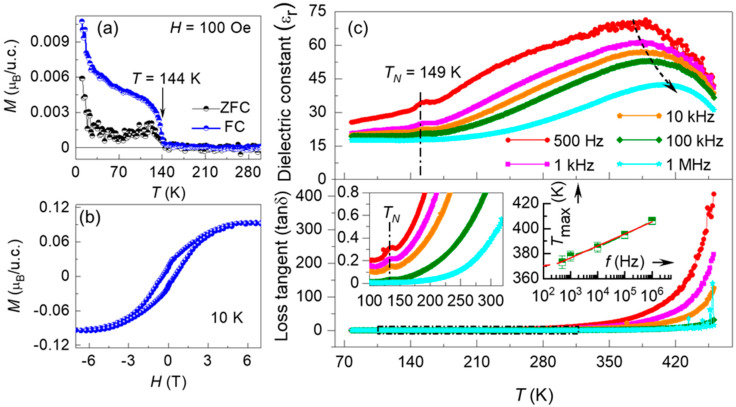
(**a**) The temperature-dependent magnetization *M*(*T*) curves measured in both the zero-field-cooled (ZFC) and field-cooled (FC) protocols, show a magnetic transition at *T* = 144 K. (**b**) The in-plane magnetic field dependence of magnetization (*M(H)*) loop of the film at 10 K. (**c**) The temperature dependence of dielectric constant ($\varepsilon_{r}$) and loss tangent (tanδ) at different frequencies. The dashed line at 149 K indicates a dielectric anomaly close to magnetic transition. The curved-dash line highlights the relaxor behavior. An enlarged view of the loss data, marked by a narrow-dashed rectangle, is represented in the left inset, showing that the dielectric anomaly can also be observed in loss tangent curves. The right inset shows a Vogel–Fulcher plot with extrapolated freezing temperature (*T*_f_ = 370 K, dashed red line), manifesting the relaxor ferroelectricity in epitaxial YCO films.

After demonstrating the synthesis of phase-pure epitaxial YCO films, the magnetic properties were examined. In bulk YCO crystals, the Cr^3+^ sublattice orders antiferromagnetically at *T*_N_ = 142 K with the moments aligned along the *c*-axis and a slight spin-canting toward the *a*-axis resulting in a net magnetic moment [24]. Figure 3a shows the temperature-dependent magnetization *M*(*T*) curves of the YCO film measured from 10 to 300 K at a magnetic field of 100 Oe applied along the in-plane direction. The *M*(*T*) curves measured in both the zero-field-cooled (ZFC) and field-cooled (FC) protocols demonstrate a divergence of the ZFC and FC moments at *T* = 144 K, thus indicating the onset of Cr magnetic ordering from the paramagnetic to the canted antiferromagnetic phase. In comparison to bulk ceramics, the epitaxial YCO film shows a slight increase in the magnetic transition temperature, which may result from epitaxial strain. We believe that a drastic increase in magnetic moment at lower temperature (<20 K) and a slightly negative magnetic moment above 144 K, are due to the STO substrate contribution, dominating the magnetic contribution of YCO which is comparatively much smaller in volume [37]. The in-plane magnetic field dependence of magnetization (*M(H)*) reveals a ferromagnetic hysteresis loop, as shown in Figure 3b, which could be a consequence of the net magnetic moment induced by canted-antiferromagnetic ordering of spins.

To investigate the possible correlation of the magnetic transitions with the dielectric behavior, the temperature-dependent dielectric studies were performed on the ~32-nm YCO film grown on conducting Nb:STO (001) substrate, using Pt top electrodes in a capacitor geometry. Figure 3c shows the dielectric constant ($\varepsilon_{r}$) and loss tangent (tanδ) of the YCO film capacitor measured at different frequencies in the span of 500 Hz to 1 MHz for the temperature range 77–460 K. A dielectric anomaly, as a kink in $\varepsilon_{r}$ and tanδ, can be observed around the magnetic ordering temperature for each frequency, in agreement with *M*(*T*) curves in Figure 3a. Interestingly, the positions of the kink in both $\varepsilon_{r}$ and tanδ do not shift with increasing frequency, which indicates that this dielectric anomaly is not associated with any relaxation phenomenon. An exponential enhancement in tanδ can be observed at higher temperatures, which could be due to the increase in the dc leakage current at high temperatures. Similar high dielectric losses and a monotonous increase in tan*δ* were reported in polycrystalline YCO films [30] and highly oriented DyCrO_3_ films [38].

Another broad peak is observed in $\varepsilon_{r}$ at *T*_C_ = 375−408 K, shifting to lower temperatures and increasing in magnitude with decreasing frequency, indicating that it could be associated with the intrinsic dielectric relaxation in YCO—a typical strong dispersion effect often attributed to the freezing-in of ferroelectric clusters [39]. A Vogel–Fulcher relationship with an extrapolated freezing temperature of around 370 K, as shown in the right inset of Figure 3c, corroborates the presence of relaxor ferroelectricity in our YCO films [40]. This observation of the dielectric phase transition agrees well with the relaxor-dielectric behavior and the related ferroelectric phase transition reported in bulk YCO powders and polycrystalline films [30]. The occurrence of a dielectric anomaly at 149 K, close to the magnetic ordering temperature, illustrates a close correlation between magnetic and dielectric properties, indicating the possibility of either a magnetodielectric effect or an additional structural phase transition in this system, which needs to be investigated in future work.

**Figure 4:**
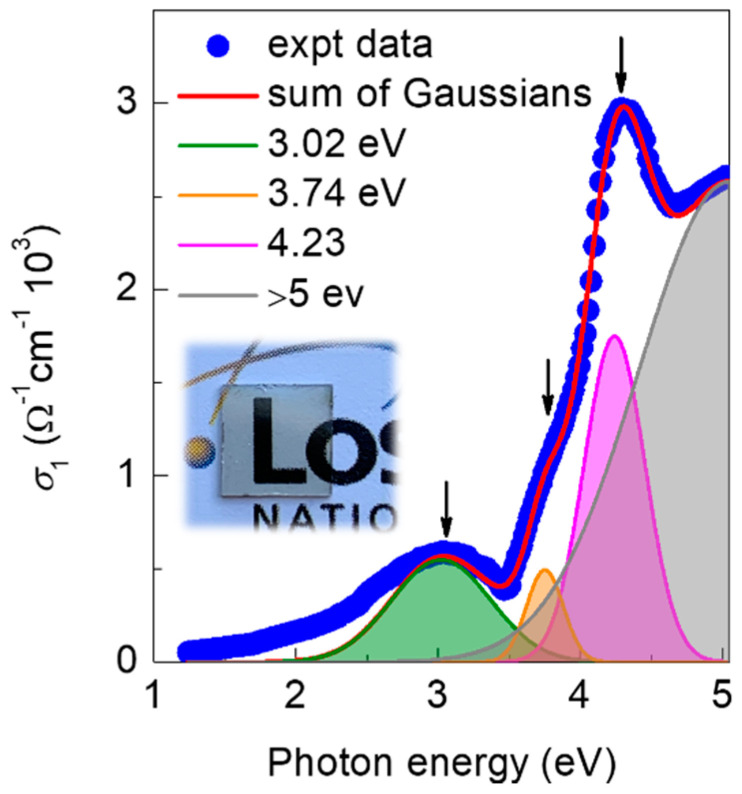
Spectroscopic ellipsometry measurement of the optical conductivity of YCO film, where the positions of all Gaussian peaks are shown with arrows. The inset shows the photograph of a 32-nm YCO film sample grown on a two-side-polished 5 × 5 × 0.5 mm^3^ STO (001) substrate.

The *R*CrO_3_ perovskites show complex optical properties in terms of multiband optical transitions [26,41]. The several optical transitions can also be observed in the 32-nm YCO films as shown in the optical conductivity spectrum [$\sigma_{1\;}\left(\omega \right)$] measured by spectroscopic ellipsometry at room temperature in Figure 4. The spectrum is well-fitted to Gaussians and the characteristic features of this spectrum include four distinct peaks at 3.02, 3.74, 4.23, and above 5 eV. Some of these bands are in good agreement with the calculated excitation energies and experimentally reported optical transitions in LCO films [26], where the four most prominent optical bands are assigned to intra-Cr *t_2g_-e_g_* (2.7, 3.6 eV), inter-Cr *t_2g_-t_2g_* (4.4 eV), and interion O 2p-Cr 3d (from ~5 eV) transitions. However, an additional peak at 3.02 eV does not match any optical transitions reported in Ref. [26]. An optical peak at ~3.3 eV was reported in LaCrO_4_ and La_2_CrO_6_ films [41], which corresponds to the green and yellow color of these samples, respectively. However, the nearly transparent nature of the YCO film (inset of Figure 4), similar to LCO films [41], and the relatively low energy of the additional peak (3.02 eV), as compared to the 3.3-eV peak of LaCrO_4_ and La_2_CrO_6_ phases, indicate that this additional peak in our sample may not be due to the presence of YCrO_4_ (Cr^5+^) or Y_2_CrO_6_ (Cr^6+^) phase inclusions. The XAS results support the argument, as no obvious deviation from Cr^3+^ can be observed in the YCO films, however, further studies are required to determine the origin of this peak. A strong optical transition can be observed to emerge at ~3.75 eV in Figure 4, which can be defined as the optical bandgap of the YCO film in agreement with the direct band gap of ~3.72 eV reported for bulk YCO [42].

## 4. Conclusions

To summarize, this work demonstrates that the structurally and stoichiometrically well-defined epitaxial YCO thin films can be grown using PLD, without any post-synthesis annealing steps. The epitaxially strained YCO films grew in a layer-by-layer fashion, giving rise to excellent crystallinity and smooth surfaces. The observation of ferromagnetic hysteresis loops at 10 K and relaxor-ferroelectric behavior at *T*_C_ = 375–408 K, ascribed the multiferroic behavior of the YCO films. A dielectric anomaly close to the magnetic ordering temperature indicates the presence of a magneto(di)electric effect in the epitaxial YCO films. These results may open up an avenue to further study the role of epitaxial strain on the on-set of magnetic and ferroelectric ordering temperatures and magnetoelectric coupling in *R*CrO_3_ material systems.
